# Editorial: Reproductive infectious diseases: matters across the spectrum of reproductive health

**DOI:** 10.3389/frph.2026.1803783

**Published:** 2026-04-07

**Authors:** Okeoma Mmeje, Tosin Goje, Khady Diouf, Craig R. Cohen

**Affiliations:** 1 Department of Obstetrics and Gynecology, University of Michigan Medical School, Ann Arbor, MI, United States; 2 Cleveland Clinic, Cleveland, OH, United States; 3 Brigham and Women’s Hospital, Boston, MA, United States; 4 Department of Obstetrics, Gynecology & Reproductive Sciences, University of California San Francisco (UCSF), San Francisco, CA, United States

**Keywords:** bacterial vaginosis, HIV prevention, reproductive infectious diseases, sexually transmitted infections, global health

Reproductive infectious diseases (RID) represent a critical yet often-neglected intersection of public health, clinical care, and health equity. RID encompasses sexually transmitted infections (STIs), bacterial vaginosis (BV), and the host of reproductive sequelae and burden on reproductive-aged women worldwide, particularly in low- and middle-income countries where access to prevention, screening, and treatment remains limited. In 2020, the World Health Organization (WHO) estimated 374 million new infections globally with four curable STIs: chlamydia (129 million), gonorrhea (82 million), syphilis (7.1 million), and trichomoniasis (156 million). The same year, approximately 540,000 women aged 15 years and older acquired HIV infection, with 20.0 million women living with HIV globally. Notably, while these infections are biologically preventable and treatable, women's reproductive health remains underfunded and marginalized within broader health systems. The intersectional impacts of social determinants of health, stigma, structural inequities, and limited healthcare access create a complex landscape wherein women experience not only acute infections but also serious long-term reproductive sequelae, including ectopic pregnancy, infertility, chronic pelvic pain, cervical cancer, and compromised quality of life.

This special issue, titled “*Reproductive Infectious Diseases: Matters Across the Spectrum of Reproductive Health*,” brings together seven peer-reviewed manuscripts that collectively advance our understanding of reproductive infectious diseases through diverse methodological approaches and international contexts. These contributions address critical knowledge gaps spanning epidemiology, clinical management, women's experiences and acceptability of interventions, and emerging prevention strategies. By examining reproductive infectious diseases across the continuum of reproductive health, from diagnosis and management to prevention and patient-centered outcomes. This collection underscores the urgent need for increased research funding, greater attention to clinical training, and policy priority in this foundational area of women's health.

## Overview of the collection

The seven articles within this special issue encompass systematic reviews, clinical trials, implementation studies, and translational investigations conducted across multiple global contexts, with particular attention to sub-Saharan Africa and other regions disproportionately affected by reproductive infectious diseases.

### Epidemiological burden and risk factors

Two articles provide a critical epidemiological context for reproductive infectious diseases. Gedfie et al. conducted a comprehensive systematic review and meta-analysis examining the prevalence and associated factors of syphilis among female sex workers in East Africa. Synthesizing data from 24 studies involving 25,979 female sex workers, the authors identified a pooled syphilis prevalence of 14.7% (95% CI: 11.1–18.4) among this key population. The analysis revealed significant geographic variation, with Ethiopia demonstrating the highest prevalence at 18.5%, underscoring regional disparities in disease burden and transmission risk. The researchers identified advanced age as a significant predictor of syphilis, emphasizing that female sex workers are not a homogeneous group and that intervention strategies must be tailored to address both structural vulnerabilities and specific demographic risk factors. This work highlights the persistent challenge of untreated syphilis, which, if left unaddressed, may result in serious complications, including adverse reproductive outcomes and congenital syphilis, diminished quality of life, and increased risk of HIV transmission.

### Cervicitis and emerging infections

Vodstrcil et al. conducted a decade-long retrospective case series analyzing infections detected among women presenting with cervicitis at Melbourne Sexual Health Centre from 2011 to 2021. Among 813 cervicitis cases, chlamydia or gonorrhea were not detected in approximately 75% of cases. These findings challenge traditional paradigms that focus on chlamydia and gonorrhea as the primary causes of cervicitis. Instead, the study revealed that BV and *Mycoplasma genitalium* emerged as alternative causes of infection. Importantly, the detection of both BV and *M. genitalium* in cervicitis cases increased significantly over the study decade, suggesting that contemporary diagnostic and management approaches must broaden beyond traditional STI testing. This epidemiologic shift has profound implications for clinical practice and highlights the evolving landscape of RID.

### HIV prevention in key populations

Nzungize and Munyaneza assessed awareness and willingness to use pre-exposure prophylaxis (PrEP) for HIV prevention among 333 female sex workers in Kigali, Rwanda. The study revealed remarkably high awareness of PrEP (81%) and willingness to use it (80%) among female sex workers. This cohort has a higher risk of HIV acquisition, given the median of seven sexual partners per week reported by their study participants. However, the analysis identified the following challenges: female sex workers reporting consistent condom use were paradoxically less likely to be aware of PrEP, and those screened for STIs were less willing to use PrEP than those not screened for STIs, highlighting the complex interaction between different prevention strategies, patient perceptions and practice. Educational attainment emerged as a significant predictor: participants with primary education were more likely to use PrEP than those without formal education. These findings underscore the need for targeted health education and STI screening initiatives that contextualize PrEP as a complementary prevention tool rather than a replacement for other protective strategies. Clinicians (i.e., nurses, physicians, and physician assistants) should share information and resources about HIV prevention with the patients they serve, which includes providing HIV prevention education and PrEP prescriptions.

### Innovative therapeutic approaches

Two articles examined novel treatment strategies for reproductive infectious disease sequelae. Adewumi et al. evaluated the acceptability of self-administered, home-delivered intravaginal 5-Fluorouracil (5-FU) cream for cervical precancer treatment among 12 women living with HIV in rural Kenya. All 12 participants reported confidence in the treatment's safety by study conclusion, and 91.7% experienced no discomfort with the vaginal applicator. Qualitatively, women appreciated the ease of use, the discreet nature of the treatment, and the privacy of the home application. The primary challenges included correctly measuring dosage, finding private space for self-administration, and maintaining condom use during treatment. Notably, all participants preferred self-administered topical therapy over traditional clinic-based ablation or excision procedures, suggesting substantial patient preference for home-based, less-invasive alternatives. This investigation demonstrates that scalable, cost-effective solutions exist for addressing the cervical precancer treatment gap in low- and middle-income countries, a gap wherein only a fraction of women identified with cervical precancer receive treatment.

Hemmerling et al. assessed the acceptability of LACTIN-V (*Lactobacillus crispatus* CTV-05), a live biotherapeutic product, among 45 young Black South African women at high risk for HIV acquisition. Participants demonstrated high adherence (80% completing all 11 doses) and strong acceptability: 88.4% were satisfied with the vaginal applicator, 95.5% confirmed ease of use, and 75% reported willingness to use the product again. Importantly, 32.5% of participants valued the ability to use the product without partners’ knowledge, and 72.1% felt that partner approval was unimportant. This highlights the significance of autonomous reproductive decision-making, particularly in contexts where gender inequity may limit women's ability to negotiate health interventions, including safer sexual practices. As a novel live biotherapeutic designed to optimize vaginal microbiota, reduce genital inflammation, and protect against HIV acquisition, LACTIN-V represents a promising prevention strategy grounded in the understanding of the vaginal microenvironment's role in HIV susceptibility.

### Patient-centered perspectives on diagnosis and health information

Two additional articles in the collection focus on the patient and public perspectives on reproductive infectious diseases. Velmahos et al. conducted a content analysis comparing symptoms of bacterial vaginosis as described on social media (Instagram, Facebook, YouTube, X/Twitter) with those reported by clinical patients. Social media significantly overemphasized burning (45% of posts vs. 21% of patients), itching (45% vs. 30% of patients), and pain (23% vs. 21% of patients) as symptoms of BV. This discrepancy highlights a critical public health concern: social media may promote self-diagnosis of vaginal conditions based on symptomatology, leading to delayed clinical evaluation and potentially inappropriate treatment. The study underscores the need for healthcare systems and providers to provide accurate, accessible digital health information that reflects clinical reality on social media.

Darivemula and Rahangdale conducted a comprehensive mini-review addressing menstrual health and menstrual equity for women living with HIV in the United States—a population intersecting multiple vulnerabilities. Despite over two decades of antiretroviral therapy transforming HIV into a chronic disease, little research has examined how women living with HIV manage menstrual health, access menstrual supplies, and navigate the intersectional stigma associated with both HIV and menstruation. The authors reviewed evidence demonstrating that menstrual irregularities are common among women living with HIV, yet gynecologic care utilization remains suboptimal, and shame related to menstruation persists across reproductive transitions. The review identifies substantial gaps in knowledge and calls for future research centering on the lived experiences of women living with HIV around the affordability and accessibility of menstrual supplies.

## Training in RID

As graduates of RID fellowship training following our residencies in Obstetrics & Gynecology, we believe that clinical and scientific training in RID is essential to addressing the complex, intersectional challenges women face globally, particularly those in marginalized and underserved populations. Enhanced training, including at the postdoctoral level, equips healthcare providers and researchers with the knowledge to navigate the evolving landscape of RID, including emerging pathogens, syndemic interactions with HIV, and innovative therapeutic and preventive approaches. By advancing clinical expertise and scientific investigation, training in RID lays the foundation for improved health outcomes, informed public health strategies, and the prioritization of women's reproductive health as a global imperative.

## Conclusion

This special issue represents a collective call to action to elevate RID as a priority research and clinical training area. The seven investigations presented herein advance our understanding of epidemiology, clinical management, acceptability, and prevention of reproductive infectious diseases, while simultaneously illuminating profound knowledge gaps and persistent inequities in care. As the global community works toward WHO's cervical cancer elimination targets and accelerated HIV prevention goals, reproductive infectious diseases must not be marginalized but instead recognized as foundational to reproductive health, quality of life, and gender equity. We hope this collection inspires future research, informs clinical practice, and ultimately drives policy changes necessary to address the burden of RID across reproductive health.

